# Effects of Hydroxylated Lecithin on Growth Performance, Serum Enzyme Activity, Hormone Levels Related to Lipid Metabolism and Meat Quality in Jiangnan White Goslings

**DOI:** 10.3389/fvets.2022.829338

**Published:** 2022-02-22

**Authors:** Hongzhi Wu, Sibo Wang, Yong Tian, Ning Zhou, Chunqin Wu, Ruiqing Li, Wenwu Xu, Tieshan Xu, Lihong Gu, Fengjie Ji, Li Xu, Lizhi Lu

**Affiliations:** ^1^College of Animal Science and Technology, Northeast Agricultural University, Harbin, China; ^2^State Key Laboratory for Managing Biotic and Chemical Threats to the Quality and Safety of Agro-Products, Institute of Animal Science & Veterinary, Zhejiang Academy of Agricultural Science, Hangzhou, China; ^3^Tropical Crop Genetic Resource Research Institute, Chinese Academy of Tropical Agricultural Sciences, Haikou, China; ^4^Wenzhou Vocational College of Science and Technology, Wenzhou, China; ^5^Institute of Animal Science & Veterinary, Hainan Academy of Agricultural Science, Haikou, China

**Keywords:** hydroxylated lecithin, Jiangnan White goslings, enzymes activity, hormone levels, lipid metabolism, meat quality

## Abstract

The objective of the present study was to evaluate the effects of hydroxylated lecithin on growth performance, serum enzyme activity, hormone levels related to lipid metabolism and meat quality in Jiangnan White goslings. Six hundred 1-day-old goslings were randomly divided into five treatments with six replicates and 20 for each replicate. The control group (CG) was fed the basal diet, while the experimental group was fed the basal diet with 50, 100, 200 mg/kg hydroxylated lecithin and 100 mg/kg soy lecithin (HLG50, HLG100, HLG200, and LG100, respectively) in the form of powder. Feed and water were provided *ad libitum* for 32 days. Compared with the CG, (a) the average daily feed intake was higher (*P* < 0.05) in HLG100, the final body weight and average daily gain were higher (*P* < 0.05), and the feed conversion ratio was lower in the HLG200; (b) the alanine aminotransferase, malate dehydrogenase, leptin, glucagon, thyroid hormone, Triiodothyronine contents in the HLG200 were lower (*P* < 0.05); (c) The breast muscle water holding capacity was higher (*P* < 0.05) in groups with hydroxylated lecithin, the breast muscle shear force and fiber diameter were lower (*P* < 0.05) in the HLG100; (d) the inositic acid, intramuscular fat, phospholipid contents were higher (*P* < 0.05), the triglyceride content was lower (*P* < 0.05) in HLG100 of the breast muscle; (e) the relative expression of sterol regulatory element-binding protein-1 genes were higher (*P* < 0.05) in the treated groups of muscles, the phosphorylase kinase gamma subunit 1 gene expression was shown an opposite trend. In comparison with LG100, (a) the feed conversion ratio was lower (*P* < 0.05) in HLG200; (b) the alanine aminotransferase and adiponectin contents were higher (*P* < 0.05), the malondialdehyde and free fatty acid contents were lower (*P* < 0.05) in HLG200; (c) the water holding capacity and intramuscular fat contents in the breast and leg muscles were higher (*P* < 0.05) in HLG200. The hydroxylated lecithin concentration of 200 mg/kg improved the growth performance, serum enzyme activity, hormone levels related to lipid metabolism, and the meat quality of Jiangnan White goslings.

## Introduction

Hydroxylated lecithin is a substance that introduces hydroxyl groups into the fatty acid double bonds of concentrated and purified soy lecithin (1, 2). It is considered a safe feed additive for its enriched content of phosphatidylcholine, lecithin, unsaturated fatty acids, choline and inositol. Moreover, lecithin is generally recognized as an essential nutrient for maintaining various physiological metabolism of animals (2–4). In livestock production, hydroxylated lecithin can be used as a nutritional supplement, emulsifier, humectant, and thickener. Its emulsification and hydrophilic properties are preferred to those in soy lecithin (5). Both hydroxylated lecithin and soy lecithin contain hydrophobic and hydrophilic groups in their molecular structures, forming stable emulsions of immiscible phases in feeds, which can be acted as surfactants (2, 6). Hydroxylated lecithin and soy lecithin can further disperse lipids entering the intestinal tract of poultry, increase the contact area between intestinal villous membrane and lipids and fat-soluble vitamins, and promote the lipids digestion, absorption and transfer (7, 8). In the initial stage of growth and development, due to the digestive system imperfect development of poultry, the secretion of bile and digestive enzymes is insufficient, making the lipid substances in the feed not be fully digested and absorbed (9–12). The emulsification function of hydroxylated lecithin and soy lecithin contributes to lipid digestion and absorption, and it was found that soy lecithin can improve the utilization of animal oil in feed for poultry (13, 14). Both hydroxylated lecithin and soy lecithin have a fatty flavor and good poultry appetizers (2). Moderate lecithin could increase poultry feed intake and daily weight gain, and decrease the feed conversion ratio of broilers; the daily weight gain was positively correlated with the soy lecithin content (15, 16). The hydroxylated lecithin and soy lecithin are rich in unsaturated fatty acids, which impact the synthesis and metabolism of fatty acids in poultry (2, 15). Lecithin can promote poultry to digest and absorb excess cholesterol under the lecithin cholesterol acyltransferase action increasing the polyunsaturated fatty acids deposition (as a percentage) (17, 18), making poultry meat more recognized by consumers. At present, the application of soy lecithin is still dominant in poultry production, and few hydroxylated lecithin applications have been reported. In this study, we based on comparing the structure and function between soy lecithin and hydroxylated lecithin, and it was hypothesized that hydroxylated lecithin would be superior to soy lecithin in promoting poultry production and was verified in this experiment.

The lipid content has a critical influence on meat quality, especially the flavor and tenderness, in poultry (19, 20). Jiangnan White goose is a fast-growing commercial goose cultivated by Jiangsu Lihua Animal Husbandry Co., Ltd. in China, and its lipid metabolism in the chick stage has a vital influence on its overall production performance. This study intends to (a) study the effects of hydroxylated lecithin on the growth performance, serum enzymes activity, hormone levels related to lipid metabolism, and meat quality of Jiangnan White gosling by adding hydroxylated lecithin to the Jiangnan White gosling diets, (b) discuss the feasibility and appropriate contents of hydroxylated lecithin in the production of Jiangnan White gosling, and (c) preliminarily explore the impact mechanism of the hydroxylated lecithin on production performance of Jiangnan White goslings, to further develop and use the hydroxylated lecithin, and accumulate experience and theoretical basis for safe and reliable additives.

## Methods and Materials

The Chinese guidelines for animal welfare conducted this study and with the animal welfare standards of the College of Animal Science and Technology, Northeast Agricultural University (NEAU-2018-0232).

### Experimental Material

Jiangnan White goslings: 1-day-old, with an average initial body weight 120.00 ± 5.00 g, provided by Jiangsu Lihua Animal Husbandry Co., Ltd.

Hydroxylated lecithin: EINECS No. 232-307-2, CAS No. 8029-76-3, with purity 99%, in the form of powder added to the basal diets, purchased from Sichuan Huayuan Shengtai Biotechnology Co., Ltd.

Lecithin: EINECS No. 232-307-2, CAS No. 8002-43-5, with purity 99%, in the form of powder added to the basal diets, purchased from Sichuan Huayuan Shengtai Biotechnology Co., Ltd.

Enzyme-linked immunoassay kits were purchased from Shanghai Sangon Biotechnology Co., Ltd.

### Experiment Design and Sample Collection

Six hundred 1-day-old female healthy Jiangnan White goslings, with body weight 120.00 ± 5.00 g, were randomly divided into five treatments with six replicates, with 20 goslings for each replicate. The control group (CG) was fed the basal diet (BD), in the granule form, while the treated groups were fed the BD with 50, 100, and 200 mg/kg hydroxylated lecithin and 100 mg/kg lecithin in the form of powder (HLG50, HLG100, HLG200, LG100, respectively). The composition (kg/100 kg) of the BD was shown in Table 1, and the fatty acid composition (g/100 g) of BD and lecithin was shown in Table 2. Feed and water were provided *ad libitum* for 32 days. The gosling house temperature was kept at 30°C in the first week, then was reduced gradually over the next 9 days to the outside temperature, the humidity at 63%, and the light was applied 24 h a day with 15-watt fluorescent lighting on 1–16 days. Moreover, the house temperature and humidity were kept pace with the outside environment, with natural light during the day and 8-watt fluorescent lighting at night on 17–32 days. The other feeding management and immunization procedures, including ventilation of the birdhouse and vaccine injection for goslings, were carried out following the goose farm guidelines.

**Table 1:** Composition (kg/100 kg) of the basal experimental diets^a^ for Jiangnan White goslings.

**Items**	**1–16 days**	**17–32 days**
**Ingredients**		
Corn	40.00	42.00
Corn protein power	8.00	7.00
Soybean oil	1.50	1.20
Soybean meal	19.00	17.00
Wheat bran	10.00	10.00
Defatted rice bran feed	16.25	17.25
Dicalcium phosphate	0.90	0.90
Limestone	3.60	3.90
Sodium chloride	0.35	0.35
Premix^b^	0.40	0.40
Total, kg	100.00	100.00
**Nutrient levels, on air-dry basis:**		
Metabolic energy^c^, ME, MJ/kg	12.04	11.04
Crude protein^d^, CP, %	20.00	18.00
Crude fiber^d^, CF, %	5.00	8.00
Calcium^d^, Ca, %	0.90	0.90
Total phosphorus^d^, P, %	0.60	0.50
Available phosphorus^d^, AP,%	0.38	0.32
Lysine^d^, Lys, %	1.10	0.90
Methionine^d^, Met, %	0.60	0.50

a *Based on the NRC (21) nutrient requirements for goslings*.

b *The premix provided the following per kg of diet: VA 15,000.00 IU, VD_3_ 5,300.00 IU, VE 100.00 mg, VK 4.00 mg, VB_1_ 2.00 mg, VB_2_ 1,200.00 mg, pantothenic acid 50.00 mg, nicotinic acid 10.25 mg, VB_6_ 3.85 mg, VB_12_ 0.10 mg, folic acid 2.00 mg, biotin 0.21 mg, VC 200.00 mg, Mn as manganese sulfate 80.00 mg, Fe as ferrous sulfate 60.00 mg, Cu as copper sulfate 20.00 mg, I as potassium iodide 3.00 mg, Se as sodium selenite 0.50 mg, Zn as zinc sulfate 100.00 mg, Choline as choline chloride 1,400.00 mg*.

c *Calculated value (21)*.

d *Analysed content*.

**Table 2:** The fatty acid composition of the basal experimental diets and lecithin for Jiangnan White goslings (g/100 g).

**Items**	**1~16 days**	**17–32 days**	**Hydroxylated lecithin**	**Soy lecithin**
Caprylic, C8:0	0.39	0.42	0	0
Capric, C10:0	0.21	0.22	0	0
Lauric, C12:0	3.94	3.91	0	0
Myristic, C14:0	1.55	1.51	0	0
Palmitic, C16:0	16.06	16.12	23.89	22.98
Palmitoleic, C16:1	0.22	0.19	2.98	4.12
Stearic, C18:0	1.81	1.74	9.87	8.74
Oleic acid, C18:1 n9c	24.88	24.91	45.98	44.91
Linoleic acid, C18:2 n6c	46.36	46.49	14.98	15.98
α-Linolenic acid, C18:3 n-3	3.06	3.05	0.46	0.42
Arachidie acid, 20:0	0.45	0.44	0.26	0.24
cis-11-Ecosenoic acid, C20:1	0.47	0.45	0	0
cis-11,14-Ecosenoic acid, C20:2	0	0	0.25	0.22
Arachidonic acid, C20:4 n-6	0	0	2.20	2.19
cis-5,8,11,14,17-Ecosenoic acid, C20:5	0	0	0.02	0.03
cis-4,7,10,13,16,19-Docosahexaenoic acid, C22:6 n-3	0	0	0.18	0.17
Behenic acid, C22:0	0.32	0.29	0	0
Lignoceric acid, C24:0	0.28	0.26	0	0

Feed intake and weight gain of goslings were recorded during the experiment period for calculating the average daily feed intake (ADFI), average daily gain (ADG), and feed conversion ratio (FCR). On the 32nd day, one gosling was randomly chosen from each replicate for sampling. Blood (10 mL) was collected with disposable vacuum blood collection tubes from the neck vein of each gosling. After resting the blood for 15 min, it was centrifuged at 3,000 rpm for 15 min to obtain the serum, divided into Eppendorf tubes and stored at −20°C refrigerators for biochemical indicators testing. Right side breast and leg muscles (10 g) were collected into 15 mL centrifuge tubes filled with formalin and stored at room temperature for section hematoxylin and eosin (HE) staining to determine the muscle fiber diameter and density. In addition, the pH value (Meat Ph direct measuring instrument PH-STAR, Germany), water holding capacity (HP607 meat hydraulic tester, China) and shear force (C-LM4 Muscle Tenderness Meter, China) were determined on breast and leg muscles. Ten gram from each muscle were collected into plastic packaging bags and stored until analyses at −20°C of the inosinic acid (HPLC, Chromaster®, Japan), intramuscular fat (Soxhlet Extractor, China), triglycerides and phospholipid (Gas Chromatograph-Mass Spectrometer, the U.S.A.). The inter-assay variation coefficients of inosinic acid, intramuscular fat, triglycerides and phospholipid were 4.66, 4.98, 4.20, 4.22%, respectively; and the intra-assay variation coefficients were 4.55, 4.66, 4.78, 4.82%, respectively. Two gram of muscles were collected into freezing tubes and stored at −80°C refrigerators for RNA extraction.

### Serum Index Determination

The kits information of serum biochemical indexes in this study was shown in Table 3. And all kits were purchased from Shanghai Sangon Biotechnology Co., Ltd.

**Table 3:** The kits information of serum biochemical indexes.

**Items**	**Abbreviation**	**Kits No**.	**Coefficients of variation**	
			**Inter-assay**	**Intra-assay**
Alanine aminotransferase	ALT	D7921044	4.39%	4.21%
Lipoprotein lipase	LPL	BC2440	4.56%	4.43%
Malate dehydrogenase	MDH	A610373	4.96%	4.95%
Adiponectin	ADPN	D711336	4.20%	4.62%
Leptin	LEP	D721019	4.22%	4.32%
Glucagon	GLC	D721189	4.15%	4.17%
Insulin	INS	D721159	4.56%	4.58%
Thyroid hormone	T4	A602869	4.98%	4.78%
Triiodothyronine	T3	HY-60029	4.80%	4.72%
Thyrotropin-releasing hormone	TRH	BK7017	4.58%	4.78%
Glucose	GLU	A501991	4.66%	4.62%
Total cholesterol	TC	D799799	4.99%	4.38%
Triglycerides	TG	D799795	4.87%	4.59%
Malondialdehyde	MDA	HY-60003	4.62%	4.54%
Free fatty acid	FFA	HY-60053	4.27%	4.29%

The total cholesterol (TC), alanine aminotransferase (ALT), glucose (GLU) were measured with a fully automatic biochemical analyzer, CG3040B, Changchun Guangji Medical Instrument Co., Ltd. In addition, the malate dehydrogenase (MDH), adiponectin (ADPN), leptin (LEP), triiodothyronine (T3), thyroid hormone (T4), thyrotropin-releasing hormone (TRH), lipoprotein lipase (LPL), free fatty acid (FFA), glucagon (GLC), insulin (INS), triglycerides (TG), and malondialdehyde (MDA) were determined with the multifunctional marker (SuPerMax 3100, China). The relevant determination operations were carried out according to the kit instructions.

### Quantification of Genes With Real-Time PCR

Fifty milligram collected breast and leg muscles with 1 mL TRIzol reagent (Invitrogen, CA) was thoroughly ground in liquid nitrogen and transferred into a 1.5 mL Eppendorf tube for further analysis. Total RNA was extracted with an RNA extraction kit (Vazyme Cat. RC112-01, 50 rxn), and levels of relative expression of sterol regulatory element-binding protein-1 (SREBP-1) and phosphorylase kinase gamma subunit 1 (PHKG1) genes were determined with real-time PCR. Primers for SREBP-1 and PHKG1 were selected according to the geese sequences registered in NCBI and designed by using Beacon Designer 7, glyceraldehyde-3-phosphate dehydrogenase (GAPDH) was used as an internal control. The primer sequences were shown in Table 4. The total real-time PCR system volume was 10 μL, and the reaction system was as follows: SYBR Green Mix 4.4 μL, upstream and downstream primers 0.3 μL each, and cDNA 5 μL. The real-time PCR procedure was as follows: 95°C for 10 min, one cycle, 95°C for 10 s, 60°C for 34 s, and 40 cycles. The Ct values of the target genes and the internal reference genes were measured, and the relative gene expression levels were calculated by the 2^−ΔΔCt^ method. Each test was repeated at least three times.

**Table 4:** Primer sequences of lipid metabolism-related genes.

**Genes**	**Primer sequences (5^**′**^-3^**′**^)**	**Product length (bp)**
SREBP-1	F:CCGCTCATCCATCAACGACA	84
	R:AGGATCGCCGACTTGTTGAG	
PHKG1	F:CCCCTTCTTCCAGCAGTACG	104
	R:AGTAAATGCGGACGGATGCC	
GAPDH	F:TAGTGAAGGCTGCTGCTGAT	102
	R:AGGTGGAGGAATGGCTGTC	

*SREBP-1, sterol regulatory element-binding protein-1; PHKG1, phosphorylase kinase gamma subunit 1; GAPDH, glyceraldehyde-3-phosphate dehydrogenase*.

### Statistical Analysis

Statistical analyses were conducted using SAS 9.4 statistics software. Data were expressed as mean ± SEM. Statistical comparisons of different treatments were performed using one-way ANOVA. Each replicate pen served as an experimental unit for the growth performance statistical analyses. One gosling from each replicate served as an experimental unit for the serum biochemistry indexes and meat quality statistical analyses. Duncan's multiple range tests determined significant differences among the treatment means at *P* < 0.05.

## Results

### Effects on the Growth Performance

As shown in Table 5, the average initial body weight (AIBW) was no significant difference (*P* > 0.05) among groups. The final body weight (FBW) and ADG were higher (*P* < 0.05) in HLG200 than those in CG and HLG50. The ADFI was higher (*P* < 0.05) in the treated groups with hydroxylated lecithin and soy lecithin than that in CG, it was higher (*P* < 0.05) in HLG100 than that in HLG200. The FCR in HLG200 was lower (*P* < 0.05) than that in CG, HLG50 and LG100.

**Table 5:** Effects of hydroxylated lecithin on growth performance of Jiangnan White goslings.

**Items**	**CG**	**HLG50**	**HLG100**	**HLG200**	**LG100**	**P Value**
AIBW, g	119 ±4.00	118 ±2.55	120 ±4.82	120 ±3.09	119 ±3.80	0.072
FBW, g	1,647 ±46.06^b^	1,659 ±31.16^b^	1,779 ±37.91^ab^	1,801 ±21.06^a^	1,691 ±22.87^ab^	0.043
ADFI, g/d	187 ±2.08^c^	196 ±1.52^ab^	198 ±2.63^a^	192 ±2.94^b^	193 ±3.72^ab^	0.034
ADG, g/d	47.32 ±1.75^b^	48.25 ±1.61^b^	51.51 ±1.81^ab^	53.03 ±1.21^a^	49.01 ±2.51^ab^	0.044
FCR, kg/kg	3.95 ±0.10^a^	4.03 ±0.16^a^	3.86 ±0.15^ab^	3.52 ±0.15^b^	3.95 ±0.14^a^	0.045

*AIBW, Average initial body weight; FBW, Final body weight; ADFI, Average daily feed intake; ADG, Average daily gain; FCR, Feed conversion ratio*.

a,b,c *Different lowercase letters in the peer data indicate that the difference is significant (P < 0.05), and no letters indicate that the difference is not significant (P > 0.05)*.

### Effects on Serum Biochemistry Indexes Related to Lipid Metabolism

#### Effects on Serum Enzymes Activity Related to Lipid Metabolism

Enzyme activity related to lipid metabolism data were summarized in Table 6. The ALT contents in treated groups were lower (*P* < 0.05) than those in CG, and the contents in LG100 were lower (*P* < 0.05) than those in groups with hydroxylated lecithin. The LPL contents in HLG100, HLG200, and LG100 were higher (*P* < 0.05) than those in CG and HLG50. The MDH contents in treated groups, especially in HLG200, were lower (*P* < 0.05) than those in CG, and the contents in HLG200 were lower than those in LG100.

**Table 6:** Effects of hydroxylated lecithin on enzymes activity related to lipid metabolism in serum of Jiangnan White goslings.

**Items**	**CG**	**HLG50**	**HLG100**	**HLG200**	**LG100**	**P Value**
ALT, U/L	18.12 ±2.10^a^	14.89 ±1.10^b^	12.01 ±1.60^c^	12.02 ±1.45^c^	9.35 ±1.02^d^	0.032
LPL, umolFFA/mL·h	1.83 ±0.01^d^	1.88 ±0.01^d^	2.48 ±0.04^c^	2.71 ±0.04^b^	2.82 ±0.02^a^	0.029
MDH, U/mL	57.69 ±10.33^a^	37.68 ±2.26^b^	33.65 ±2.07^c^	20.18 ±6.06^e^	30.56 ±4.21^d^	0.033

*ALT, Alanine aminotransferase; LPL, Lipoprotein lipase; MDH, Malate dehydrogenase*.

a,b,c,d,e *Different lowercase letters in the peer data indicate that the difference is significant (P < 0.05)*.

#### Effects on Serum Hormone Levels Related to Lipid Metabolism

The effects of hydroxylated lecithin on hormone levels related to lipid metabolism were shown in Table 7. The ADPN contents in treated groups were higher (*P* < 0.05) than those in CG, and it was higher (*P* < 0.05) in HLG200 than that in LG100. The LEP contents were lower (*P* < 0.05) in HLG100, HLG200, and LG100 than those in CG and HLG50. The GLC contents in treated groups were lower (*P* < 0.05) than those in CG, and it was lower (*P* < 0.05) in HLG200 than that in LG100. The INS contents in treated groups were higher (*P* < 0.05) than those in CG, and it was higher (*P* < 0.05) in LG100 than that in HLG100. The T4 and T3 contents in treated groups were lower (*P* < 0.05) than those in CG, and they were lower (*P* < 0.05) in HLG200 than those in LG100. The TRH contents in treated groups were lower (*P* < 0.05) than those in CG, and it was lower (*P* < 0.05) in HLG100 than that in LG100.

**Table 7:** Effects of hydroxylated lecithin on hormone levels related to lipid metabolism in serum of Jiangnan White goslings.

**Items**	**CG**	**HLG50**	**HLG100**	**HLG200**	**LG100**	**P Value**
ADPN, mg/L	8.99 ±1.46^d^	12.58 ±1.20^c^	16.26 ±2.10^ab^	16.44 ±2.21^a^	15.44 ±1.22^b^	0.035
LEP, ng/mL	4.72 ±0.10^a^	4.67 ±0.14^a^	3.54 ±0.20^b^	3.32 ±0.22^b^	3.57 ±0.21^b^	0.044
GLC, pg/mL	111 ±10.20^a^	95.15 ±4.76^b^	85.59 ±4.39^c^	75.29 ±4.69^d^	85.06 ±3.31^c^	0.026
INS, uIU/mL	8.64 ±1.04^d^	10.83 ±1.09^c^	11.02 ±0.15^bc^	12.75 ±0.05^b^	14.77 ±1.02^a^	0.033
T4, ng/mL	24.44 ±1.56^a^	21.30 ±1.57^b^	19.32 ±1.05^c^	17.61 ±1.01^d^	21.11 ±1.05^b^	0.039
T3, ng/mL	1.84 ±0.31^a^	1.14 ±0.01^b^	1.11 ±0.01^b^	1.03 ±0.03^c^	1.13 ±0.01^b^	0.046
TRH, pg/mL	19.60 ±1.17^a^	12.03 ±1.60^d^	14.81 ±1.10^c^	16.71 ±1.18^b^	17.52 ±1.04^b^	0.034

*ADPN, Adiponectin; LEP, Leptin; GLC, Glucagon; INS, Insulin; T4, Thyroid hormone; T3, Triiodothyronine; TRH, Thyrotropin-releasing hormone*.

a,b,c,d *Different lowercase letters in the peer data indicate that the difference is significant (P < 0.05)*.

#### Effects on Serum Other Biochemical Indicator Levels Related to Lipid Metabolism

The other biochemical indicator levels related to lipid metabolism data were summarized in Table 8. The GLU contents in treated groups were lower (*P* < 0.05) than those in CG, and it was lower (*P* < 0.05) in HLG50 and HLG200 than that in LG100. The TC and TG contents in treated groups were lower (*P* < 0.05) than those in CG, and the TG content was lower (*P* < 0.05) in HLG200 than that in LG100. The MDA contents in treated groups were lower (*P* < 0.05) than those in CG, and it was lower (*P* < 0.05) in groups with hydroxylated lecithin than that in LG100.The FFA contents in treated groups were higher (*P* < 0.05) than those in CG, and it was lower (*P* < 0.05) in groups with hydroxylated lecithin than that in LG100.

**Table 8:** Effects of hydroxylated lecithin on other biochemical indicator levels related to lipid metabolism in serum of Jiangnan White goslings.

**Items**	**CG**	**HLG50**	**HLG100**	**HLG200**	**LG100**	**P Value**
GLU, mmol/L	14.60 ± 1.50^a^	8.36 ± 1.50^c^	11.52 ± 1.47^b^	8.35 ± 1.51^c^	11.43 ± 1.56^b^	0.044
TC, mmol/L	5.45 ± 0.34^a^	4.72 ± 0.46^b^	3.73 ± 0.24^c^	3.67 ± 0.23^c^	3.57 ± 0.26^c^	0.047
TG, mmol/L	1.66 ± 0.05^a^	1.48 ± 0.04^b^	1.39 ± 0.00^bc^	1.31 ± 0.00^c^	1.44 ± 0.01^b^	0.043
MDA, nmol/mL	4.95 ± 0.10^a^	4.53 ± 0.08^c^	4.17 ± 0.31^d^	3.63 ± 0.22^e^	4.72 ± 0.12^b^	0.029
FFA, mmol/L	0.50 ± 0.01^e^	0.53 ± 0.01^d^	0.57 ± 0.02^c^	0.60 ± 0.01^b^	0.63 ± 0.01^a^	0.026

*GLU, Glucose; TC, Total cholesterol; TG, Triglycerides; MDA, Malondialdehyde; FFA, Free fatty acid*.

a,b,c,d,e *Different lowercase letters in the peer data indicate that the difference is significant (P < 0.05)*.

### Effects on Meat Quality

#### Effects on Meat Physiological Indexes

As shown in Table 9, the breast muscle pH value was higher (*P* < 0.05) in LG100 than that in HLG100 and HLG200. Leg muscle pH value was lower (*P* < 0.05) in treated groups than that in CG, and it was lower (*P* < 0.05) in LG100 than that in HLG100. The breast muscle water holding capacity was higher (*P* < 0.05) in groups with hydroxylated lecithin than that in CG and LG100. The leg muscle water holding capacity was higher (*P* < 0.05) in HLG100 than that in CG and LG100. The breast muscle shear force was lower (*P* < 0.05) in HLG100 than that in CG and LG100, and leg muscle shear force was lower (*P* < 0.05) in the treated groups than that in CG (*P* < 0.05). The breast muscle fiber diameter was lower (*P* < 0.05) in HLG100 than that in CG and LG100 (*P* < 0.05), while the leg muscle fiber diameter was lower (*P* < 0.05) in HLG100 than that in CG. The breast muscle fiber density was higher (*P* < 0.05) in HLG200 and LG100 than that in CG, and the leg muscle fiber density was higher (*P* < 0.05) in the treated groups than that in CG.

**Table 9:** Effects of hydroxylated lecithin on meat muscle water holding capacity and shear force of Jiangnan White goslings.

**Items**	**CG**	**HLG50**	**HLG100**	**HLG200**	**LG100**	**P Value**
BMpH	5.84 ±0.15^ab^	5.84 ±0.16^ab^	5.71 ±0.04^b^	5.71 ±0.08^b^	5.95 ±0.15^a^	0.046
LMpH	5.90 ±0.12^a^	5.74 ±0.13^b^	5.76 ±0.11^b^	5.55 ±0.11^c^	5.54 ±0.10^c^	0.039
BMWHC, %	14.56 ±0.41^c^	15.00 ±0.73^b^	15.80 ±0.61^a^	15.09 ±0.22^b^	14.80 ±0.20^c^	0.042
LMWHC, %	13.34 ±0.41^d^	15.04 ±0.04^c^	16.34 ±0.09^a^	16.31 ±0.63^b^	15.04 ±0.09^c^	0.036
BMSF, N	58.10 ±2.74^b^	55.07 ±1.73^b^	50.72 ±2.38^c^	50.32 ±2.78^c^	61.28 ±2.03^a^	0.043
LMSF, N	41.45 ±2.35^a^	33.48 ±2.88^c^	36.45 ±2.15^b^	36.73 ±2.43^b^	37.57 ±1.14^b^	0.046
BMFD, μm	25.25 ±2.00^a^	26.57 ±2.04^a^	20.88 ±2.75^b^	16.29 ±1.50^c^	16.48 ±1.82^c^	0.042
LMFD, μm	92.79 ±5.76^a^	91.90 ±5.87^a^	81.65 ±3.61^b^	65.39 ±4.54^d^	71.47 ±6.52^c^	0.036
BMFY, N/mm^2^	1120 ±29.50^b^	1063 ±22.56^b^	1057 ±17.13^b^	1358 ±32.27^a^	1434 ±47.85^a^	0.048
LMFY, N/mm^2^	110 ±11.63^d^	125 ±11.01^c^	127 ±17.20^c^	167 ±20.72^b^	237 ±29.60^a^	0.034

*BMpH, Breast muscle pH value; LMpH, Leg muscle pH value; BMWHC, Breast muscle water holding capacity; LMWHC, Leg muscle water holding capacity; BMSF, Breast muscle shear force; LMSF, Leg muscle shear force; BMFD, Breast muscle fiber diameter; LMFD, Leg muscle fiber diameter; BMFY, Breast muscle fiber density; LMFY, Leg muscle fiber density*.

a,b,c,d *Different lowercase letters in the peer data indicate that the difference is significant (P < 0.05)*.

#### Effects on Meat Biochemistry Indexes

The meat biochemistry indexes were summarized in Table 10. In the breast muscle, the inositic acid and intramuscular fat contents were higher (*P* < 0.05) in HLG100 than those in CG and LG100. The triglyceride content was lower (*P* < 0.05) in HLG100 than that in CG and LG100. Phospholipid content was significantly higher (*P* < 0.05) in the treated groups than that in CG. In the leg muscle, the inositic acid, intramuscular fat and phospholipid contents were higher in HLG100 than those in CG. The triglyceride content was lower (*P* < 0.05) in HLG100 and LG100 than that in CG.

**Table 10:** Effects of hydroxylated lecithin on muscle inosinic acid and other indicators of Jiangnan White goslings.

**Items**	**CG**	**HLG50**	**HLG100**	**HLG200**	**LG100**	**P Value**
Breast muscle inosinic acid, mg/g	2.04 ± 0.10^d^	2.05 ± 0.10^d^	2.80 ± 0.11^a^	2.48 ± 0.11^b^	2.41 ± 0.10^c^	0.036
Breast muscle intramuscular fat, %	3.13 ± 0.15^c^	3.48 ± 0.01^a^	3.49 ± 0.01^a^	3.49 ± 0.01^a^	3.42 ± 0.10^b^	0.041
Breast muscle triglycerides, mg/g	3.12 ± 0.11^a^	3.14 ± 0.10^a^	2.75 ± 0.03^c^	3.14 ± 0.10^a^	2.85 ± 0.05^b^	0.043
Breast muscle phospholipid, mg/g	0.98 ± 0.02^d^	1.05 ± 0.03^c^	1.31 ± 0.10^a^	1.15 ± 0.04^b^	1.30 ± 0.10^a^	0.037
Leg muscle inosinic acid, mg/g	2.40 ± 0.02^d^	2.56 ± 0.06^bc^	2.75 ± 0.11^a^	2.52 ± 0.06^c^	2.70 ± 0.09^ab^	0.032
Leg muscle intramuscular fat, %	3.00 ± 0.02^b^	3.15 ± 0.11^a^	3.17 ± 0.10^a^	3.16 ± 0.10^a^	3.15 ± 0.11^a^	0.046
Leg muscle triglycerides, mg/g	3.16 ± 0.11^a^	2.95 ± 0.13^ab^	2.76 ± 0.05^c^	2.93 ± 0.10^b^	2.77 ± 0.06^c^	0.036
Leg muscle phospholipid, mg/g	1.04 ± 0.09^c^	1.16 ± 0.02^b^	1.22 ± 0.03^a^	1.22 ± 0.01^a^	1.21 ± 0.02^a^	0.043

a,b,c,d *Different lowercase letters in the peer data indicate that the difference is significant (P < 0.05)*.

#### Effects on Genes Relative Expression in Breast and Leg Muscles

**Figure 1:**
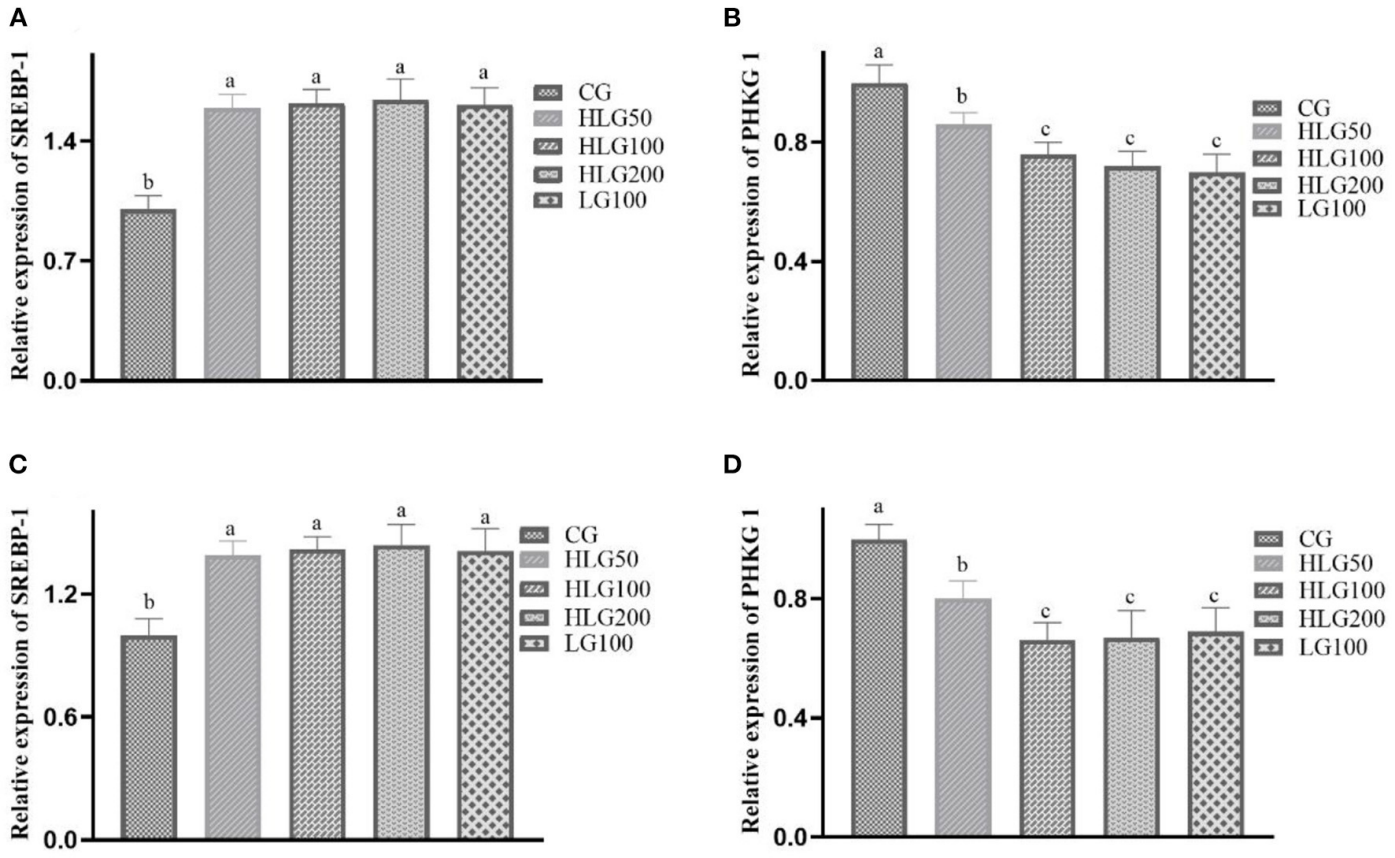
The relative expression levels of genes relative to meat quality in breast and leg muscle of Jiangnan White goslings. The data in CG, HLG50, HLG100, HLG200, and LG100 in breast muscle were **(A)** SREBP-1 gene: 1.00 ± 0.10^b^, 1.59 ± 0.08^a^, 1.62 ± 0.08^a^, 1.64 ± 0.12^a^, 1.61 ± 0.10^a^, respectively; **(B)** PHKG 1 gene: 1.00 ± 0.10^a^, 0.86 ± 0.04^b^, 0.76 ± 0.04^c^, 0.72 ± 0.05^c^, 0.70 ± 0.06^c^, respectively; The data in CG, HLG50, HLG100, HLG200, and LG100 in leg muscle were **(C)** SREBP-1 gene: 1.00 ± 0.10^b^, 1.39 ± 0.07^a^, 1.42 ± 0.06^a^, 1.44 ± 0.10^a^, 1.41 ± 0.11^a^, respectively; **(D)** PHKG 1 gene: 1.00 ± 0.10^a^, 0.80 ± 0.06^b^, 0.66 ± 0.06^c^, 0.67 ± 0.09^c^, 0.69 ± 0.08^c^, respectively.

As shown in Figure 1, the relative expression levels of the SREBP-1 gene in the treated groups were higher (*P* < 0.05) than those in the CG of breast and leg muscles. The PHKG 1 gene relative expression in HLG100, HLG200, and LG100 was lower than that in the CG of breast and leg muscles.

## Discussion

### Effects on Growth Performance

Growth performance is an indispensable indicator of poultry growth status under different conditions (22, 23). The utilization of unsaturated fatty acids in poultry is much higher than that of saturated fatty acids (24, 25). Hydroxylated lecithin and soy lecithin provide nutrients and many unsaturated fatty acids to poultry (15).

In this study, the results of adding 50, 100, and 200 mg/kg of hydroxylated lecithin and 100 mg/kg of soy lecithin to the basal diet of goslings showed that the FBW was significantly higher in HLG200 than that in the CG and HLG50. The ADFI in the treated groups with hydroxylated lecithin and soy lecithin was considerably higher, the ADG in HLG200 was substantially higher than that in the CG, but the FCR was shown an opposite trend. Ren et al. (26) found that hydroxylated lecithin could significantly improve the average daily weight gain and average daily feed intake of three-yellow chickens. Gu and Li (27) found that soy lecithin had a significant effect on the average daily weight gain and the feed-to-weight ratio of piglets and could significantly reduce the feeding costs. Wang et al. (28) found that phosphatidylcholine, one of the main components of soy lecithin and hydroxylated lecithin, could dramatically improve the production performance of broiler chicks. Mandalawi et al. (29) found that soy lecithin could significantly improve the feed-to-egg ratio of laying hens. Furthermore, the results in this study were consistent with the above results, indicating that both hydroxylated lecithin and soy lecithin could improve the growth performance of goslings and the effect of hydroxylated lecithin is better than that of soy lecithin. Huang et al. (30) found no significant effect of soy lecithin on the performance of broiler chicks, which is not consistent with the results of this experiment, which may be related to the experimental animal species, conditions, soy lecithin ingredients and supplementation.

The hydroxylated lecithin and soy lecithin are effective emulsifiers, and both can promote the absorption of fat in feed for poultry (2, 15). Sibbald and Kramer (31) found that soy lecithin could improve the animal fats utilization in poultry, and Dei et al. (32) found that soy oil could improve the fats digestibility coefficient of broiler chickens. In this study, the hydroxylated lecithin significantly reduced goslings' FCR, which may be associated with the increasing cholecystokinin secretion stimulated by the hydroxylated lecithin in the small intestine that can delay the gastrointestinal emptying. This physiological process can extend the feed residence time in the gastrointestinal tract so that nutrients can be fully digested and absorbed in goslings.

### Effects on Serum Biochemistry Indexes Related to Lipid Metabolism

#### Effects on Serum Enzymes Activity Related to Lipid Metabolism

The ALT activity can indirectly reflect the fatty acid synthesis state, and it is the most sensitive indicator of damage to hepatocytes. Liver damage can directly cause lipid metabolism disorders in animals (33). Liang et al. (34) found that the ALT activity increase in crucian carp indicated that the hepatocytes oxidative damage, which would cause the lipid metabolism disorder in the body of crucian carp. Song et al. (35) found that the ALT activity decrease in cells was accompanied by a reduction of intracellular fatty acid deposition, which indicates that ALT can effectively respond to lipid metabolism in the body and signal the formation or treatment for fatty liver and other pathologies. In this study, the serum ALT activity was significantly decreased, indicating that the hydroxylated lecithin and soy lecithin can effectively maintain the healthy state of liver cells and reduce the ALT activity, thus enabling the lipid metabolism process to proceed smoothly. In addition, the decrease of triglycerides and increase of free fatty acids in the serum of goslings in this study also verified the effect of alanine aminotransferase on the body. Further, it reflected that a healthy liver is an indispensable guarantee for lipid metabolism in the body.

LPL is one of the lipid metabolism limiting enzymes in poultry and has a vital influence on body fat deposition and intramuscular fat content (36). LPL catalyses the breakdown of TG into fatty acids and monoglycerides in the poultry, providing the organism with the energy needed for various metabolism (37). He et al. (38) found that increasing LPL mRNA and protein levels in hepatocytes could accelerate intracellular lipid metabolism. Kaneko et al. (39) found that LPL in the body of lean juvenile red seabream showed an increasing trend followed by a decreasing trend with the fat level increase in the diet. However, if the fat level in the diet were too high, the LPL activity would be inhibited, and the accumulation of fat deposition in the liver would harm the health of lean juvenile red seabream. In this study, the serum LPL activity in treated groups except for HLG50 was significantly increased, indicating that hydroxylated lecithin and soy lecithin could improve the LPL activity in goslings, which played a regulatory role in the fatty acids synthesis and metabolism. Furthermore, the serum GLU, TG and TC of goslings in this study were lower than those in the CG, which also verified to a certain extent the regulatory role of LPL on lipid metabolism in the organism.

MDH is a vital enzyme in the malate shuttle system of poultry to ensure that NADH generated by glycolysis in the cytosol enters the mitochondria for complete oxidation to produce energy, which plays an indispensable role in the complete oxidation or mutual transformation of nutrients in poultry (40, 41). In this study, the MDH activity in goslings was significantly decreased, indicating that the hydroxylated lecithin and soy lecithin reduced the oxaloacetate content, a raw material in the tricarboxylic acid cycle, thus inhibiting the fatty acid oxidation process and enhancing the deposition of lipids in poultry.

#### Effects on Serum Hormone Levels Related to Lipid Metabolism

ADPN is an endogenous bioactive cytokine and hormone secreted by adipocytes in poultry (42). After binding to the receptor, ADPN has a vital role in regulating the sugars and lipids metabolism in the organism, and it can improve insulin sensitivity, accelerate glucose metabolism and promote lipids metabolism in poultry (43). De Koster et al. (44) found that the ADPN could reduce the levels of β-hydroxybutyric acid, non-lipidated fatty acids and triacylglycerol in periparturient cows, i.e., ADPN could promote the lipids metabolism. Tahmoorespur et al. (45) found that the expression levels of adiponectin and its receptors were significantly increased when broilers needed much energy for body growth and development underfeeding restriction and other conditions, i.e., ADPN has an essential regulatory role in lipid metabolism and energy metabolism in poultry. In this study, the serum lipocalin content in treated groups was significantly increased due to the vigorous growth of goslings requiring more energy to prompt the lipids metabolism. The results in this study are consistent with the above studies.

LEP in poultry can regulate lipid metabolism in the organism by exciting the sympathetic nervous system (46, 47). Li et al. (48) found that LEP could inhibit lipid synthesis in hepatocytes, promote hepatocyte lipolysis and lipid deposition in Siniperca chuatsi under a high-fat feeding environment; Lin et al. (49) found that LEP could improve insulin resistance and increase insulin sensitivity in rats, and Steinberg Greg and Dyck (50) found that the LEP content decrease accelerated the lipid metabolism in obese rats caused by high-fat diets. In this study, except for HLG50, the LEP contents in the treated groups were significantly decreased, indicating that the hydroxylated lecithin and soy lecithin can reduce LEP content to promote lipid metabolism, making the goslings produce foraging behavior, which was consistent with the above study results.

GLC has a decisive role in promoting glycogenolysis and gluconeogenesis, which can cause a significant increase in blood GLC. In contrast, INS promotes the GLC uptake and utilization by tissue cells and stimulates glycogen synthesis inhibiting gluconeogenesis, causing the blood GLU decrease in poultry (51–53). Gallagher et al. (54) found that improving INS sensitivity and reducing INS resistance can regulate the metabolic level of adipocytokines in patients with metabolic syndrome. Adeva-Andany et al. (55) found that fat oxidation metabolism was inhibited when INS level increased, and fat anabolism was inhibited if GLC level increased in animals. In this study, the GLC contents in the groups treated with hydroxylated lecithin and soy lecithin were significantly lower than those in CG. In contrast, the INS contents were showed an opposite trend, indicating that the hydroxylated lecithin and soy lecithin regulated the lipid anabolism by increasing the INS content and decreasing the GLC content in goslings.

The TRH, a 3-peptide hormone secreted by the hypothalamus, can stimulate the pituitary gland thyrotropin-secreting cells to secrete the thyrotropic hormone (56). Thyroid hormones mainly include T4 and T3, which promote gluconeogenesis and hepatic glycogen synthesis in poultry (57). Sinha et al. (58) found that excessive T4 caused more fat breakdown than fat synthesis, leading to decreased cholesterol levels and weight loss in mice. Schering et al. (59) found that T4 in beef cattle could promote fatty acid oxidation and accelerate TC degradation if the level of T4 in beef cattle was within a reasonable range, the lipid synthesis and decomposition were in a dynamic balance, and the blood lipids were maintained at an average level. Ge et al. (60) found that the high energy level diets would reduce T3 in broiler breeders and thus promote lipid metabolism. In this study, the treated groups' TRH, T3, and T4 contents were significantly decreased, resulting from hydroxylated lecithin and soy lecithin regulating the TRH content, handling the T4 and T3 contents to complete the lipid synthesis and metabolism regulation in goslings.

### Effects on Meat Quality

The poultry meat quality can generally be evaluated by pH value, water holding capacity and shear force (61). Various aspects influence muscle pH value changes, and the direct influence factor is muscle glycolysis. The pH value visualizes muscle acidity and alkalinity and can directly impact muscle tenderness and drip loss (61). In this study, the BMpH in HLG100 and HLG200 was lower, the LMpH in the treated groups with hydroxylated lecithin and soy lecithin was lower, indicating that hydroxylated lecithin could reduce the pre-growth muscles pH value of geese. Poultry muscle water holding capacity has a critical impact on meat processing. If the muscle water holding capacity is strong, the muscle itself can be well-preserved nutrients and flavor components in the production and processing to enhance the muscle quality. Lee et al. (62) found that the higher muscle water holding capacity can increase the muscle flavor of broiler breasts. In this study, the BMWHC was higher in HLG100 and HLG200, and the LMWHC was significantly higher in the treated groups, indicating that hydroxylated lecithin and soy lecithin can improve the breast and leg muscle water holding capacity of goslings, thus improving its meat quality. Stefania et al. (63) found that muscle tenderness improved with reduced muscle shear force in pigs. In this study, the BMSF in HLG200 was lower, and LMSF in the treated groups was more downward, indicating that the hydroxylated lecithin and soy lecithin could improve the breast and leg muscle tenderness of goslings.

The muscle fiber small-diameter and large-density can improve the meat tenderness in poultry (64). In this study, the BMFD and LMFD in HLG100, HLG200, and LG100 were lower, the BMFY and LMFY were higher in HLG200 and LG100, indicating that the hydroxylated lecithin and soy lecithin could improve the meat tenderness goslings, which echoes the decrease in breast and leg muscle shear force above to increase the meat tenderness.

The inosinic acid has a vital influence on the meat flavor in poultry (65). Zhang et al. (66) found that the inosinic acid was correlated with the meat flavor of broilers, and Wang et al. (65) found that different feed additives could determine the meat freshness grade by affecting the muscle inosinic acid content. In this study, the higher breast muscle inosinic acid content in HLG100, HLG200, and LG100, and the higher leg muscle inosinic acid content in treated groups, indicating that the hydroxylated lecithin and soy lecithin could improve the meat flavor of goslings. Intramuscular fat includes lipid droplets within muscle fibers and fat deposited between muscle fibers and fiber bundles (67). The intramuscular fat content in muscle critically influences the meat flavor and tenderness. Zhang et al. (68) found that the expression of the SREBP-1 gene, PHKG 1 gene, fatty acid synthase gene, hormone-sensitive lipase gene, and lipoprotein lipase gene could regulate intramuscular fat deposition, thus improving the pork flavor and tenderness. Yang et al. (69) found that the meat tenderness of Peking duck could be enhanced with the intramuscular fat content increase. In this study, the intramuscular fat content in goslings' breast and leg muscles was increased, indicating that the hydroxylated lecithin affects the intramuscular fat deposition in the muscles, improving the meat tenderness flavor of goslings. And the results of SREBP-1 gene and PHKG 1 gene relative expression in HLG100 also validated the above results at the RNA levels. TG, the most abundant lipids, can be broken down to provide energy for poultry (70). The phospholipid is an indispensable part of the biological membrane structure (70). Therefore, TG and phospholipids can affect the meat quality of poultry in different ways. In this study, the breast and leg muscle TG contents in HLG100 and LG100 were lower; and the phospholipids content in treated groups was higher, indicating that the hydroxylated lecithin could improve the meat quality by decreasing the triglycerides content and increasing the phospholipids content.

## Conclusion

The effects of hydroxylated lecithin on growth performance, serum enzyme activity, hormone levels related to lipid metabolism and meat quality of Jiangnan White goslings in the different treatment groups were assessed. The final body weight, average daily gain, feed conversion ratio, alanine aminotransferase, malate dehydrogenase, leptin, glucose, triiodothyronine, thyroid hormone, malondialdehyde, free fatty acid, breast muscle water holding capacity, leg muscle water holding capacity, intramuscular fat contents, relative expression levels of the sterol regulatory element-binding protein-1 and phosphorylase kinase gamma subunit 1 genes in HLG200 were improved compared with the CG and/or LG100. Overall, the hydroxylated lecithin concentration of 200 mg/kg improved the growth performance, serum enzyme activity, hormone levels related to lipid metabolism, and the meat quality of Jiangnan White goslings. Therefore, hydroxylated lecithin can be widely used as a safe and reliable additive in livestock production.

## Data Availability Statement

The original contributions presented in the study are included in the article/supplementary material, further inquiries can be directed to the corresponding authors.

## Ethics Statement

The animal study was reviewed and approved by the Chinese guideline for animal welfare and with the animal welfare standards of the College of Animal Science and Technology, Northeast Agricultural University (NEAU-2018-0232).

## Author Contributions

HW and SW prepared the manuscript and collected some data. YT, NZ, CW, RL, WX, TX, LG, and FJ collected the samples. LL and LX were responsible for the design and direction of the experiment. All authors have read and agreed to the published version of the manuscript. All authors contributed to the article and approved the submitted version.

## Funding

This work was supported by the China Agriculture Research System of MOF and MARA, China Scholarship Council (CSC) National Public Graduate Program for Building Highly Qualified Universities, and Wenzhou New poultry variety breeding cooperation group project (2019ZX005).

## Conflict of Interest

The authors declare that the research was conducted in the absence of any commercial or financial relationships that could be construed as a potential conflict of interest.

## Publisher's Note

All claims expressed in this article are solely those of the authors and do not necessarily represent those of their affiliated organizations, or those of the publisher, the editors and the reviewers. Any product that may be evaluated in this article, or claim that may be made by its manufacturer, is not guaranteed or endorsed by the publisher.
